# Biopolymer‐Based Multilayer Microparticles for Probiotic Delivery to Colon

**DOI:** 10.1002/adhm.202102487

**Published:** 2022-03-03

**Authors:** Sepehr Talebian, Timothy Schofield, Peter Valtchev, Aaron Schindeler, John M. Kavanagh, Qayyum Adil, Fariba Dehghani

**Affiliations:** ^1^ School of Chemical and Biomolecular Engineering The University of Sydney Sydney NSW 2006 Australia; ^2^ Nano Institute (Sydney Nano) The University of Sydney Sydney NSW 2006 Australia; ^3^ Centre for Advanced Food Engineering The University of Sydney Sydney NSW 2006 Australia; ^4^ Bioengineering & Molecular Medicine Laboratory The Children's Hospital at Westmead and the Westmead Institute for Medical Research Westmead NSW 2145 Australia; ^5^ PharmaCare Laboratories 18 Jubilee Ave Warriewood NSW 2102 Australia

**Keywords:** colon, drug delivery, hydrogels, microencapsulation, probiotics

## Abstract

The potential health benefits of probiotics may not be realized because of the substantial reduction in their viability during food storage and gastrointestinal transit. Microencapsulation has been successfully utilized to improve the resistance of probiotics to critical conditions. Owing to the unique properties of biopolymers, they have been prevalently used for microencapsulation of probiotics. However, majority of microencapsulated products only contain a single layer of protection around probiotics, which is likely to be inferior to more sophisticated approaches. This review discusses emerging methods for the multilayer encapsulation of probiotic using biopolymers. Correlations are drawn between fabrication techniques and the resultant microparticle properties. Subsequently, multilayer microparticles are categorized based on their layer designs. Recent reports of specific biopolymeric formulations are examined regarding their physical and biological properties. In particular, animal models of gastrointestinal transit and disease are highlighted, with respect to trials of multilayer microencapsulated probiotics. To conclude, novel materials and approaches for fabrication of multilayer structures are highlighted.

## Graphical abstract

The health merits of probiotics are compromised, mainly due to major reduction in their viability during storage and gastrointestinal transit phase. Biopolymeric multilayer microparticles provide enhanced level of protection for the encapsulated probiotics, which lead to significant improvements in their therapeutic potential.

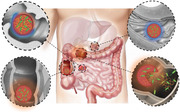

## 1 Introduction

Over the last decade, probiotics have gained immense popularity among the general public; however, their health benefits remain contraversial.^[^ ^1^ ^]^ While some claim probiotics can significantly affect human health, others emphasize that such gains are highly influenced by heterogeneity related to probiotic strains, individuals, and their microbiome.^[^ ^2^ ^]^ Despite multiple randomized clinical trials showing alleviation of symptoms associated with gastrointestinal conditions,^[^ ^3^, ^4^, ^5^, ^6^ ^]^ there is no general agreement on the usefulness of probiotic treatments. Despite their yet unproven therapeutic merits, the lack of efficacy could be correlated with suboptimal delivery of probiotics.

**Figure 1:**
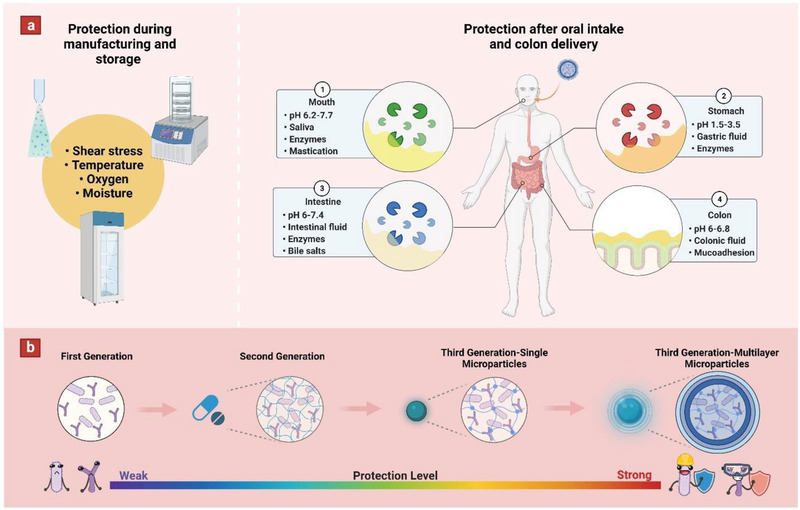
Schematic showing a) various factors affecting probiotic viability during production, storage, and after oral intake. b) Evolution of probiotic delivery systems from first generation to third generation multilayer microparticles and their corresponding protection level.

Two widely used and studied lactic acid bacterial genera that are usually represented in probiotic products are *Lactobacillus* and *Bifidobacterium*. Along these lines, various species of these two genera such as *Bifidobacterium breve*, *Bifidobacterium longum* (*B. longum*), *Lactobacillus fermentum* (*L. fermentum*), *Lactobacillus plantarum* (*L. plantarum*), and *Lactobacillus rhamnosus* (*L. rhamnosus*) have been successfully incorporated into commercial products in the form of beads, granules, capsules, and tablets.^[^ ^7^ ^]^ The preferred health benefits of probiotics are primarily expressed if the appropriate amounts of colony‐forming units (CFU) colonize at the lower gastrointestinal tract and colon.^[^ ^8^, ^9^, ^10^ ^]^ However, there are some challenges for probiotic formulation due to physicochemical conditions in gastrointestinal tract. Shear stress, enzymatic degradation, acidic pH (gastric and bile acids), mineral ions, anaerobic conditions, and mucoadhesion as probiotics pass through the mouth, stomach small intestine, and colon (**Figure** **1**a).^[^ ^11^, ^12^ ^]^ Shear stress from mechanical stimulation during mastication may also damage the surface of the beads and the peristalsis of the esophagus may stimulate the breakdown of the bead morphology.^[^ ^13^ ^]^ Moreover, enzymes within the saliva including mucin and amylases can interact with the beads to rehydrate and potentially weaken the surface bonding.^[^ ^14^ ^]^ Within the stomach the pH ranges from 1 to 3 or higher after a meal in combination with the presence of mineral ions and enzymes (lipase and protease) that further interact with the bead surface.^[^ ^15^, ^16^, ^17^ ^]^ The beads will then pass through the small intestine and interact with bile acids and enzymes at a pH of 6.8–7.4.^[^ ^16^ ^]^ Ideally, when the beads reach the colon, they de‐encapsulate and the probiotics then compete with the resident bacteria to adhere to the mucosal wall. Probiotic viability can also be affected by the manufacturing process and storage conditions.^[^ ^18^ ^]^ Thus, it is pivotal to design a probiotic delivery system to withstand exposure to oxygen, moisture, shear stress, high temperature, enzyme, and pH in gastric track.

Various strategies have been practiced overtime to preserve the viability of probiotic strains. First‐generation probiotics were defined as live and/or lyophilized bacterial cells lacking a capsule or microcapsule.^[^ ^19^ ^]^ The survival rate of these probiotic products under the acidic and alkaline environment of the gastrointestinal tract was significantly low within the range of 7–30%. Moreover, this generation has a limited shelf life as evident from short viability of only 2 weeks for *B. longum* in fermented dairy products.^[^ ^20^ ^]^ To address these shortcomings, the second‐generation of probiotics incorporated lyophilized microorganisms in polymeric capsules or tablets with synthetic, semisynthetic, or natural fillers. The addition of sodium alginate as tablet filler was found to increase the survival of probiotic bacteria to 90% or more during passage through the gastrointestinal tract in experimental models, while shelf life increased to 6 months.^[^ ^21^ ^]^ However, such simple encapsulation strategies remain susceptible to rapid one‐time release of probiotic strains in the proximal gastrointestinal tract before reaching the colon. Consequently, as shown in Figure 1b, third‐generation probiotics were developed, featuring microencapsulation to tune their viability and release mechanisms.^[^ ^22^ ^]^ Microencapsulation is achieved by physically entrapping the probiotics within a dense biopolymeric (natural or synthetic) network, which can minimize molecular mobility during storage time and thus reduce the degradation rate. An extensive list of biopolymers used for microencapsulation of probiotics are provided in the following published review papers.^[^ ^18^, ^23^, ^24^ ^]^ Biopolymers for encapsulation of probiotics must be biocompatible, biodegradable, processable, and neutral to probiotics.^[^ ^25^ ^]^ Moreover, they must have the ability to completely release the loaded probiotics or to allow controlled or/and targeted release of the probiotics under certain conditions. On this notion both natural (proteins or carbohydrates) and synthetic (polymethacrylate‐based copolymers or cellulose derivatives) biopolymers with characteristics such as enzyme‐, redox‐, and pH‐sensitivity were successfully employed to microencapsulate probiotics and subsequently deliver them to the colon.^[^ ^26^ ^]^ Compared to synthetic biopolymers that have a simpler and more random structure, the natural biopolymers are complex molecular assemblies that mimic macromolecules of the native extracellular environment. Consequently, natural biopolymers are less toxic, nonimmunogenic, noncarcinogenic, and nonthrombogenic. On the other hand, synthetic biopolymers have a low cost and high thermal and mechanical properties that distinguishes them from their natural counterparts.^[^ ^27^ ^]^

The biopolymeric microencapsulated probiotics are generally divided into two major categories being single‐ and multi‐layer microparticles. Single layer microparticles are generally composed of a semipermeable, spherical, thin, and strong membranous wall that retains the probiotics within the structure. The nutrients and metabolites can diffuse through the semipermeable membrane easily. The membrane acts as a barrier to cell release and minimizes contamination. The encapsulated probiotic is released by several mechanisms through this membrane either by its mechanical rupture, dissolution, melting, or through diffusion.^[^ ^28^ ^]^ Microencapsulation in these systems is achieved using techniques such as spray drying, freeze‐drying, foam drying, and fluidized bed drying.^[^ ^29^ ^]^ Consequently, the release rate of probiotics under gastrointestinal conditions was affected by polymeric formulation, processing parameters, and microcapsule's physical properties (such as porosity and swelling ratio).^[^ ^30^ ^]^ However, these technologies induce bacterial stress, and protective strategies are therefore needed to ensure their survival. Alternatively, emulsion and extrusion techniques were utilized in which cross‐linking of the polymeric network was achieved after either suspension in oil or dropping into a cross‐linker, respectively.^[^ ^31^ ^]^ These two techniques allowed for optimization of the cross‐linking density in the polymeric network through either physical or chemical bonding. Subsequently, viability of the encapsulated probiotics against various factors was affected by the nature and density of these cross‐linking agents.^[^ ^32^ ^]^ Despite such favorable attributes, single layer microparticles fell short of providing complete protection for the probiotics during storage, as well as exposure to gastrointestinal fluids and digestive enzymes. To address this shortfall, multilayer microparticles were produced which are composed of two or more layers from different biopolymers; one of them forms the inner core and the others make the outer layers or the shells. Such design facilitates tuning the composite material which possesses properties not achievable by the individual biopolymers of the core and the shells.^[^ ^33^ ^]^ Based on the combination of the core and the shells biopolymers, multilayer microparticles can be categorized into three groups being two‐, three‐, and four‐layer microparticles. Ideally, the multilayer microcapsule system should maintain the stability of the probiotics during storage, protect them from the harsh conditions in the upper GIT, release them throughout the colon, and then promote their ability to colonize the mucosal surfaces.^[^ ^11^ ^]^ While the choice of biopolymers in each layer are important, the interactions between these layers play an imperative role in ensuring maximum protection for the encapsulated probiotics. Simultaneously, polymeric network features such as cross‐linking nature and density, as well as degradation behavior are governing factors over performance of multilayer systems. While increased cross‐linking density could improve the protection against harsh conditions, it could jeopardize timely release of probiotics in the colon. Another criterion to be considered is the choice of fabrication method, as it dictates the morphology and possible number of layers that can be applied over the probiotics. Consequently, when designing biopolymer‐based multilayer microparticles various factors must be considered before finalizing a formulation.

In this review, we summarized recent advances in biopolymer‐based multilayer microencapsulated probiotics including encapsulation methods and polymer chemistry, and finally examine the effect of encapsulation. Specifically, we highlighted the role of multilayer microparticles for circumventing common issues associated with shelf life and delivery of probiotics to the colon. We also discussed the biomedical application of these formulations, in animal models, to alleviate symptoms associated with various gastrointestinal conditions. Last, future trends in development of multilayer microencapsulated probiotics are explored.

## 2 Methods for Multilayer Microencapsulation: Operating Parameters, Advantages, and Disadvantages

**Figure 2:**
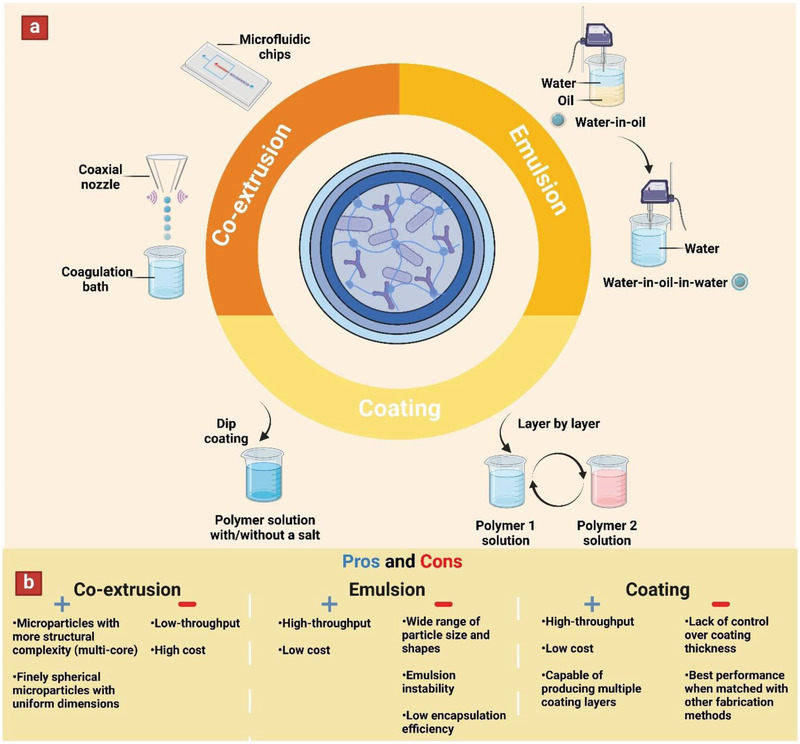
Schematic illustration showing a) various fabrication methods utilized for making multilayer microparticles (including co‐extrusion, emulsion, and coating techniques), as well as b) Advantages and disadvantages of each fabrication method.

Biopolymer‐based multilayer microparticles provide a unique opportunity to address some of the commonly associated issues with probiotic delivery to colon. However, before focusing on application of such microparticles, it is essential to discuss the various fabrication methods that can be used to produce multilayer structures, and further examine their correlation with particle properties. Microparticle characteristics such as size, morphology, and structure are important features for loading of bioactive agents.^[^ ^34^ ^]^ The fabrication methods that are commonly used for designing multilayer particles include co‐extrusion, emulsion, and coating techniques (**Figure** **2**a). In this section, the benefits and limitations of each technique is described as briefly outlined in Figure 2b.

### 2.1 Co‐Extrusion

Co‐extrusion consists of two different polymeric solutions being pumped through two nozzles to produce microparticles with core–shell morphologies. Then the formed droplets are collected in a coagulation bath. In this system, the capsules are produced using vibrational technologies,^[^ ^35^, ^36^, ^37^, ^38^, ^39^ ^]^ or microfluidic chips^[^ ^40^, ^41^, ^42^ ^]^ to break the laminar liquid jet into equal‐sized droplets that are collected at the end of the process. When using vibrational technologies, size of microcapsules is a function of multiple factors including vibrational parameters, viscosity of the encapsulant polymeric mixture, coagulation bath concentration, and the size of the extrusion nozzle.^[^ ^43^ ^]^

Unlike vibrational technologies, in microfluidic systems the properties of immiscible fluids are exploited at a microscale to generate and manipulate droplets. Accordingly, droplet templates from the microfluidics system are obtained due to the dragging force which has an intensity that is higher than that of the viscosity force.^[^ ^44^ ^]^ The droplet formation modes can be applied to various channel geometries including cross‐flow, coflow, and flow‐focusing.^[^ ^45^ ^]^ Particularly, microparticles with greater structural complexity (multicore, higher‐order) can be achieved by incorporating additional compartments in the microfluidic chip. In microfluidic systems, the size of microcapsules is mainly affected by capillary size, flow conditions, and the cross‐linking time.^[^ ^46^ ^]^

Generally, vibrational technologies hold an advantage over microfluidic systems in that they allow high‐throughput production of microcapsules, facilitating scale‐up and future industrial‐scale production.^[^ ^47^ ^]^ Conversely, the main advantage of microfluidic approaches is the fine control over the droplet‐formation process, which can be precisely tuned via designing microfluidic channels with specified geometries and controlling flow rates.^[^ ^48^ ^]^ These two techniques were prevalently used for fabrication of two‐layer microparticles containing a core and a shell compartment. Overall, microfluidic systems can produce smaller microparticles (5–500 µm) when compared to vibrational technologies (110–1250 µm).^[^ ^49^ ^]^ At the same time, both techniques allow fabrication of finely spherical microparticles with homogenous dimensions, imposing low particle size coefficient of variation ranging between 1% and 2%.^[^ ^48^ ^]^

### 2.2 Emulsion

Conventionally, emulsion consists of two phases that include a dispersed phase which contains probiotic‐polymer aqueous suspension and an oil (vegetable oil/mineral oil or organic solution) that is considered as a continuous phase. The emulsion is generally prepared by homogenizing the mixture with the aid of surfactants using a homogenizer. The particles are formed within the oil phase by the application of a cross‐linking agent or cooling process to insolubilize the water‐soluble polymer.^[^ ^50^ ^]^ Multilayer microcapsules are normally produced by means of multiple emulsions. Accordingly, a water‐in‐oil (W_1_/O) emulsion is produced by homogenizing water, oil, and an oil‐soluble emulsifier, and then a water‐in‐oil‐in‐water (W_1_/O/W_2_) emulsion is produced by homogenizing the W_1_/O emulsion with an aqueous solution containing a water‐soluble emulsifier.^[^ ^51^, ^52^, ^53^, ^54^, ^55^, ^56^, ^57^, ^58^ ^]^ Last, the particles are cross‐linked and subsequently centrifuged or filtrated.

The size and stability of droplets at both stages can be controlled by means of changing the emulsifier type, emulsifier concentration, homogenization conditions (e.g., energy intensity and duration), rate of addition of cross‐linking agents, and water to oil ratio.^[^ ^59^ ^]^ The double emulsion technique is advantageous mainly because the encapsulated probiotic can be protected from biological and environmental factors by incorporating them in the inner water phase (W_1_), thereby isolating them from other water‐soluble ingredients by means of the intermediate oil layer. However, double emulsion is associated with problems such as emulsion instability, vigorous stirring, random incorporation of the probiotics into the capsules, production of a wide range of particle size and shapes, and low encapsulation efficiency.^[^ ^18^ ^]^ Noting, most of these issues were resolved by using microfluidic generation of controllable double emulsions.^[^ ^60^ ^]^ W_1_/O/W_2_ systems are common modality for fabrication of three‐layer microparticles. Overall, emulsification is capable of yielding droplets smaller than 100 µm, and this ultimately determines the final size of the microcapsules (ranging between 25 and 2000 µm).^[^ ^61^ ^]^

### 2.3 Coating Techniques

One approach to apply extra protective layers to a microparticle is the application of a coating polymeric layer on the microcapsules surface. The coating layer can be applied in three different manners: 1) the coating polymer is incorporated into cross‐linking bath of an extrusion or spray set‐up, and hence it is acting as both a cross‐linking agent and a coating layer for the resulting microparticles;^[^ ^62^, ^63^, ^64^, ^65^ ^]^ 2) the coating polymer is applied onto the surface of a pre‐cross‐linked microparticles;^[^ ^66^, ^67^, ^68^, ^69^, ^70^, ^71^, ^72^, ^73^, ^74^, ^75^, ^76^, ^77^ ^]^ and last, 3) combination of both these techniques.^[^ ^78^ ^]^ Alternatively, the preformed microparticles (or pristine bacterial suspension) can be coated with multiple layers of oppositely charged polymers through a layer by layer electrostatic deposition technique.^[^ ^79^, ^80^ ^]^ The outermost coating layer itself can also be cross‐linked (chemically or physically) to bestow better sealing effect to the microparticles.^[^ ^81^ ^]^ Depending on the coating modality, this technique is capable of fabricating two‐, three‐, and even four‐layer microparticles to endow further protection for the encapsulated probiotics.

Overall, the coating layers will decrease the capsule's permeability leading to reduced exposure of probiotics to oxygen (during storage), as well as improving the viability of thermostable probiotics at low pH. These coatings can be engineered to develop multifunctional particles with unique properties such as mucoadhesion, and responsive release of micronutrients. Main advantage of coating techniques is their simplicity and high throughput, which is essential to their industrial applications. However, one of their main shortcomings is the lack of control over the thickness of the coating layer.^[^ ^82^ ^]^ This has been partially addressed via layer‐by‐layer deposition technique, as the number of deposited layers can determine the ultimate coating dimensions. Using this technique allows addition of coating layers with dimensions ranging from 40 to 3 µm.^[^ ^61^ ^]^

In summary, different techniques can be used to design particles with desirable properties, morphology, and one or more protective layers. While co‐extrusion and microfluidic systems allow fabrication of two‐layer polymeric microparticles, double emulsion enables three‐layer microparticles with two polymeric layers and an intermediate oily layer. The oily phase can limit humidity absorption by the particles, hence increase the viability of probiotics during storage. Furthermore, multimodal therapeutics can be achieved by incorporating hydrophobic antioxidant agents into the oily layer, which can remarkably enhance their bio‐accessibility.^[^ ^83^, ^84^ ^]^ Coating techniques are the most versatile modality as they can be combined with other microencapsulation techniques to create two‐, three‐, or four‐layer microparticles with fine tuning the composition of each layer and their intermolecular interactions. Therefore, by forming robust polymeric networks, maximum protection for the probiotics can be achieved. In the following section, we will have a closer look at various polymer chemistries that were used in the context of multilayer microparticles and assess their performance in maintaining the viability of probiotics for the purpose of delivery to colon.

## 3 Biopolymer‐Based Multilayer Microparticles for Probiotic Delivery

There is strong evidence that multilayer microcapsules can contribute to the better protection of probiotics against multiple factors associated with storage and colon delivery of these therapeutics. In this section, an overview is given for various formulations that are practiced within each multilayer design to maximize protection of the encapsulated probiotics. There is a correlation between the number of layers and the protection level for the probiotics. Hence, we have categorized these multilayer microparticles based on their number of layers, being two‐, three‐, and four‐layer microparticles.

### 3.1 Two‐Layer Microparticles

These microparticles often include a polymeric/oily core that is covered by a polymeric shell. In fact, each compartment itself can be composed of multiple constituents including complementary polymers, nutrients, prebiotics, and cryoprotectants to impose further protection to the encapsulated probiotics. When complementary polymers are used, they are bound to have ionic interactions/hydrogen bonding with the primary polymer to add further cross‐linking to the polymeric network. The probiotics are either encapsulated in the core compartment or conversely in the shell. Clearly, incorporation in the shell confers a less degree of protection, however, nutrients/prebiotics is usually incorporated in the core to support the viability of the probiotics. Based on the composition of core and shell compartments, the two‐layers microparticles are divided into three different categories: 1) single component core + single component shell, 2) multicomponent core + single component shell, and last, 3) single component core + multicomponent shell (**Table** **1**).

**Table 1:** Summary of two‐layer microparticles for probiotic delivery

Fabrication technique	Core component/s	Shell component/s	Probiotic strain	Results	Ref.
Extrusion + Dip coating	Ca cross‐linked alginate	Chitosan	*Saccharomyces boulardii*	Enhanced storage viability (120 days at 30 °C), and gastrointestinal resistance compared to single alginate particles.	^[^ ^85^ ^]^
Emulsion + Dip coating	Ca cross‐linked alginate oligosaccharide	Chitosan oligosaccharide	*Bifidobacterium longum*	20% reduction in cell viability after in vitro gastrointestinal testing, compared to 40% for alginate containing particles. Improved growth and increased amount of probiotics in the intestine (mice), leading to reduced pathogenic bacteria.	^[^ ^86^ ^]^
Emulsion + Dip coating	Ca cross‐linked alginate	Chitosan	*Bifidobacterium longum*	50% improvement in viability loss after 180 days at 25 °C, compared to free cells. Only 30% reduction in viability after gastrointestinal testing, compared to 100% reduction for single alginate particles.	^[^ ^70^ ^]^
Electrospray + Dip coating	Ca cross‐linked alginate	Chitosan	*Lactobacillus plantarum*	Excellent gastric‐mucoadhesion after 120 min of washing with simulated gastric fluid (Ex vivo‐porcine gastric mucosa).	^[^ ^88^ ^]^
Emulsion + Dip coating	Ca cross‐linked alginate	Chitosan	*Enterococcus* *sp*. (Fluorescent labeled)	6 Logs higher cell concentration in the gastrointestinal tract (In vivo‐ Black‐footed abalone), compared to conventionally fed animals.	^[^ ^68^ ^]^
Extrusion + Dip coating	Ca cross‐linked polyacrylate‐grafted alginate	Chitosan	*Lactobacillus plantarum* (Fluorescent labeled)	Cell viability increased by ninefold after incubation in simulated intestinal and colon fluids. 5 Logs and 2 Logs higher cell viability in ileum and colon (In vivo Wistar rats), respectively (compared with alginate particles).	^[^ ^71^ ^]^
Extrusion + Dip coating	Ca cross‐linked alginate	Chitosan cross‐linked with Na‐tripolyphosphate	*Lactobacillus plantarum*	Lowest reduction in cell viability after simulated gastrointestinal conditions, compared to single alginate and chitosan‐coated alginate.	^[^ ^90^ ^]^
Emulsion + Dip coating	Ca cross‐linked alginate	PVP‐*co*‐DMAEMA	*Lactobacillus plantarum*	Significantly higher cell viability after 24 h in osmotic stress, compared to not coated particles	^[^ ^92^ ^]^
Extrusion + Dip coating	Ca cross‐linked alginate	Zein	*Bifidobacterium bifidum*	Maximum viable cell count with minimum log reduction after exposure to SGJ and SIF. Highest cell viability after 32 days storage at 4 °C.	^[^ ^63^ ^]^
Extrusion + Dip coating	Ca cross‐linked alginate + various grades of inulin	Chitosan	*Lacticaseibacillus casei*	Microparticles containing long‐chain inulin had the lowest reduction in viability upon gastrointestinal and bile salts testing, when compared to pristine alginate–chitosan formulations.	^[^ ^72^ ^]^
Electrospray + Dip coating	Ca cross‐linked alginate + long‐chain inulin or resistant starch	Chitosan	*Lactobacillus plantarum* or *Bifidobacterium lactis*	Microcapsules containing resistant starch had a better cell viability under gastrointestinal conditions. On the other hand, inulin‐containing microcapsules improved the survival of cells during 90 days of storage (at 25, 4, or −18 °C).	^[^ ^100^ ^]^
Extrusion + Dip coating	Ca cross‐linked alginate + xanthan gum	Chitosan	*Lactobacillus plantarum*	Higher cell viability after exposure to simulated gastric fluid when compared to chitosan‐coated alginate particles.	^[^ ^102^ ^]^
Extrusion + Dip coating	Ca cross‐linked alginate + pea protein	Chitosan	*Lacticaseibacillus rhamnosus* and *Lactobacillus helveticus*	No loss in viable cell counts observed after treatment with simulated gastrointestinal conditions in samples stored under different temperatures (4 or 22 °C).	^[^ ^103^ ^]^
Emulsion + Dip coating	Ca cross‐linked pectin + green tea extract	Whey protein isolate	*Lactobacillus helveticus*	After exposure to simulated gastric juice, the mean survival rate of cells in core–shell microparticles containing 1000 µg mL^−1^ GTE was significantly higher (100%) than the one of cells in core–shell formulations without GTE (69%).	^[^ ^67^ ^]^
Electrospray + Dip coating	Ca cross‐linked alginate	Egg albumen + stearic acid	*Lactobacillus acidophilus*	Increase in relative proportion of stearic acid led to enhanced encapsulation efficiency, and significant improvement in viability of encapsulated cells exposed to the moist‐heat treatment	^[^ ^65^ ^]^
Co‐extrusion	Sunflower oil	Ca cross‐linked alginate + Shellac	*Lactobacillus acidophilus*	Higher cell viability after both storage and exposure to gastrointestinal conditions when compared to particles without shellac.	^[^ ^108^ ^]^
Co‐extrusion	Sunflower oil or Coconut fat	Ca cross‐linked alginate + Shellac	*Lactobacillus paracasei*	Core–shell particles containing alginate–shellac blend in the shell and coconut fat in the effectively protected the encapsulated probiotic under simulated gastrointestinal conditions, as compared to particles with sunflower oil.	^[^ ^35^ ^]^

#### 3.1.1 Single Component Core + Single Component Shell

**Figure 3:**
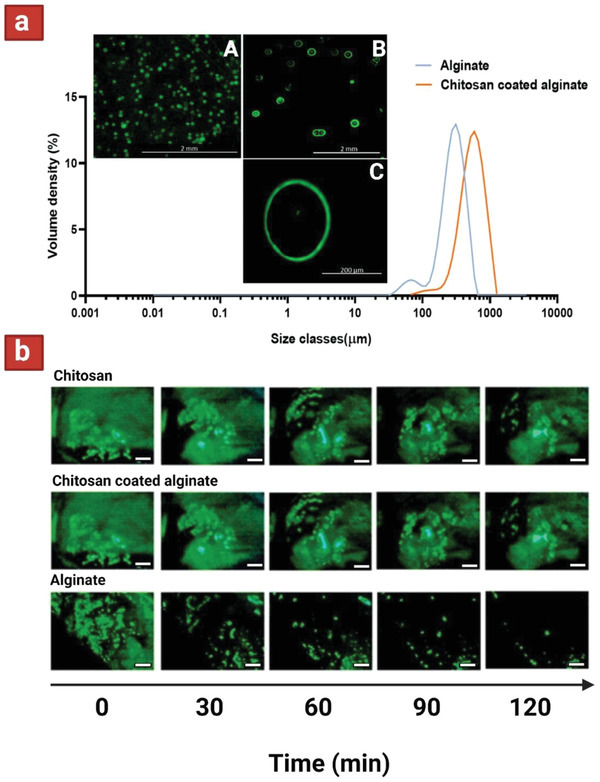
a) Particle size distribution of alginate and chitosan‐coated alginate microcapsules. Inserts show fluorescent microscopy images representing alginate (A), and chitosan coating layer on alginate (B,C). Scale bars showing 2 mm and 200 µm for low and high magnification images, respectively. b) Fluorescence images showing retention of each variation of microcapsules on porcine gastric mucosa after the indicated time of washing with simulated gastric fluid (0.2% w/v NaCl, pH 2). Scale bar is 1000 µm. Reproduced with permission.^[^ ^88^ ^]^ Copyright 2021, Elsevier.

A common approach is to use opposingly charged polymers in the core and shell compartments. Charged interactions ensure a secure and even coating, even when common materials are used such as alginate and chitosan.^[^ ^66^, ^85^, ^86^, ^87^ ^]^ An example of this was the emulsification of free cells (*B. longum*) in an alginate hydrogel that were dip‐coated in a chitosan solution.^[^ ^70^ ^]^ This 36 µm coating slightly decreased the encapsulation yield, but dramatically increased survivability in simulated gastric fluid (SGF) (pH 2.5) and simulated intestinal fluid (SIF) with bile salts (pH 6.8). An electrospraying technique was used to encapsulate *L. plantarum* in calcium cross‐linked (via CaCl_2_) alginate microparticles, which were then dip‐coated by chitosan to yield two‐layers structures.^[^ ^88^ ^]^ The mean sizes of the whole particle population were estimated to be 309 and 607 µm for uncoated alginate and coated alginate particles, respectively (**Figure** **3**a). The mucoadhesion of these microparticles (containing FITC‐dextran) were subsequently evaluated using an in vitro fluorescence imaging‐based flow‐through test on ex vivo porcine gastric epithelial mucosa. It was shown that around 62% and 32% of remaining fluorescence intensity could still be observed after 60 and 120 min of washing with SGF (pH 2), respectively. On the other hand, more rapid removal was observed in case of pure alginate, especially over the last 50 min of the 2 h experiment (Figure 3b).

A challenge with interpreting data from probiotic encapsulation studies is a heavy reliance on in vitro assays in the absence of animal or clinical data. To this end, alginate microparticles with a chitosan coating have been tested in an abalone model, featuring encapsulation of fluorescently tagged *Enterococcus sp*.^[^ ^68^ ^]^ In this study, the bacterial load in the gastrointestinal tract was considerably higher with encapsulation.

The attributes of single‐component core/shell particles can be further tuned by adjusting the ionic interactions between the chitosan and alginate, or by introducing further cross‐linking into the chitosan coating layer. The former is achieved by modifying the alginate backbone with further carboxylic acid groups to endow more negative charge into the core. This allows higher protonation of alginate in the gastric environment, hence achieving stronger ionic interactions with the chitosan shell.^[^ ^71^ ^]^ Inducing further chitosan cross‐linking features in a study employing Na‐tripolyphosphate to create encapsulated microparticles of *L. plantarum*.^[^ ^89^ ^]^ This increased resistance to exposure to simulated intestinal juice. Adjusting the molecular weight and concentration of chitosan coating can be used as an alternative option to control the ionic interactions between chitosan and alginate.^[^ ^90^ ^]^

While chitosan remains one of the most used shell materials, it may result in allergic or immunomodulatory effects in some consumers. As a result, coating of alginate microcapsules with more biocompatible polycations such as buttermilk protein,^[^ ^91^ ^]^ zein,^[^ ^63^ ^]^ and poly(1‐vinylpyrrolidone‐*co*‐2‐dimethylaminoethyl methacrylate)^[^ ^92^ ^]^ emerged as alternative options with promising results. Alginate microparticles can also be coated with polyanions,^[^ ^93^ ^]^ or neutrally charged oligosaccharides^[^ ^94^ ^]^ on the basis of hydrogen bonds. Although these interactions are not as strong as ionic bonding, they still provide a certain level of protection for the encapsulated probiotics.

To conclude, within this category of simple two‐layered microparticles, a polyanion is predominantly used in the core alongside a probiotic while a polycation is used in the shell to leverage from ionic bonds between these two compartments. Furthermore, using chemical modification, it is possible to further tune the negative charge in the core or the positive charge in the shell, which contributes to stronger ionic interactions leading to improved protection of the encapsulated probiotics. Noting, there have been few cases in which a polycation was used in the core while a polyanion was utilized as a shell.^[^ ^73^, ^81^, ^95^ ^]^ However, these formulations are not as useful as their counterparts in protecting the encapsulated probiotics, mainly because ionic cross‐linking of the polyanionic shell (via divalent cations) is susceptible to the acidic pH of the stomach.

#### 3.1.2 Multicomponent Core + Single Component Shell

Multicomponent core + single component shell is similar to the previous class, except extra components are added to the core compartment of the microparticles. The extra components in the core could be prebiotics, cryoprotectants, complementary polymers, or a combination of these. While prebiotics are added as nutrients for maintaining probiotics viability, complementary polymers are incorporated to reinforce the core's polymeric network by means of physical/chemical interactions. Chitosan‐coated alginate microparticles are effective in protecting the probiotics, and consequently multiple studies focused on enhancing their performance by adding extra components to the alginate core. Accordingly, in separate studies various prebiotics and cryoprotectants (such as inulin,^[^ ^69^ ^]^ resistant starch,^[^ ^96^ ^]^ waxy starch,^[^ ^97^ ^]^ trehalose,^[^ ^98^ ^]^ and glycerol^[^ ^99^ ^]^) have been added to the alginate core to enhance probiotic viability.

In a noteworthy study, various inulin grades (native, long‐chain, and short‐chain) were separately incorporated into alginate (containing *Lactobacillus casei* [*L. casei*]), and then chitosan‐coated alginate microparticles were fabricated through extrusion/dip coating.^[^ ^72^ ^]^ Compared to pristine alginate–chitosan formulations, *L. casei* encapsulated in core–shell microparticles containing long‐chain inulin had the lowest reduction in viability upon incubation in simulated gastric juice (pH 1.5) and simulated intestinal juice (pH 7.25). These effects most likely result from slower fermentation of long‐chain inulin, and its higher stability in the range of pH and ionic strength of the human gastrointestinal tract.

Given the diversity of prebiotics, researchers also tried to compare their performance against each other when incorporated into chitosan‐coated alginate microparticles.^[^ ^74^ ^]^ Accordingly, *L. plantarum* and *Bifidobacterium lactis* (*B. lactis*) were co‐encapsulated separately with either inulin (long‐chain) or resistant starch in alginate–chitosan‐coated microcapsules fabricated by electrospray technology.^[^ ^100^ ^]^

**Figure 4:**
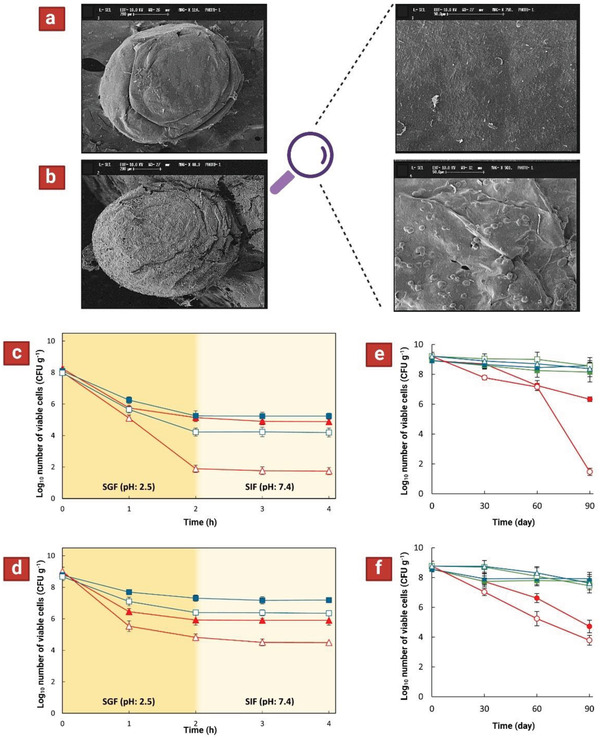
Shape (left—scale bars represent 200 µm) and surface morphology (right—scale bars represent 50 µm) of microcapsules containing a) bacteria–inulin and b) bacteria–resistant starch. Survival curve under simulated gastrointestinal conditions for probiotics encapsulated in chitosan‐coated Ca–alginate particles containing c) inulin or d) resistant starch. *L. plantarum* ATCC 8014 (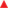) or *B. lactis* (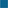). Survival of *L. plantarum* ATCC 8014 under 90 days of storage conditions (at 25 °C (●), 4 °C (■), or −18 °C (▲)) in two‐layer microparticles containing e) inulin or f) resistant starch. Solid and open symbols represent encapsulated and free (control) cells, respectively. Reproduced with permission.^[^ ^100^ ^]^ Copyright 2019, Elsevier.

Resistant starch contains highly compacted granules that poorly gelatinize, and thus limit water penetration and accessibility of digestive enzymes (**Figure** **4**a,b). On the other hand, inulin enhanced the cell survival during storage by reducing moisture content and water activity.^[^ ^101^ ^]^ Incorporation of resistant starch into microcapsules containing *B. lactis* considerably improved bacterial survival under SGF (pH 2.5), and SIF (pH 7.4). In general, microcapsules containing resistant starch revealed a better performance in maintaining the viability of probiotics under gastrointestinal conditions than those contained inulin (Figure 4c,d). However, inulin‐containing microcapsules improved the survival of *lactobacilli* more efficiently than starch‐containing coating during storage for 90 days at 25, 4, or −18 °C) (Figure 4e,f).

Aside from addition of prebiotics, introduction of complementary polymers into the alginate core is also practiced for enhancing performance of chitosan‐coated alginate microcapsules. For this purpose, both anionic (xanthan gum)^[^ ^102^ ^]^ and cationic (pea protein)^[^ ^103^ ^]^ polymers were added to the alginate core to increase the polymeric network density. *L. plantarum* incorporated into chitosan‐coated alginate–xanthan gum microparticles had a higher viability after exposure to simulated gastric juice (pH 1.8) when compared to that of chitosan‐coated alginate microparticles. However, subsequent incubation in SIF (pH 6.8) did not show any significant difference between the two groups. The enhanced acid‐resistance of these microparticles was attributed to improved bonding between the core components themselves, as well as with the chitosan shell.

Other than alginate, pectin (an anionic polysaccharide) has also been used as the main core polymer in the context of two‐layer microparticles with “multicomponent core + single component shell".^[^ ^77^, ^104^, ^105^ ^]^ In these cases, pectin microparticles are normally coated with whey protein isolate (a cationic polypeptide). For instance, *Lactobacillus helveticus* and green tea extract (GTE‐natural antioxidant) were co‐encapsulated in calcium pectinate microparticles via emulsification and subsequently dip‐coated with whey protein isolate.^[^ ^67^ ^]^ Following exposure to simulated gastric juice (pH 2), the mean survival rate of cells in core–shell microparticles containing 1000 µg mL^−1^ GTE was significantly higher (100%) than those cells in core–shell formulations without GTE (69%). In conclusion, addition of extra components to the core compartment could be an efficient option to enhance viability of encapsulated probiotics in the core–shell formulations.

#### 3.1.3 Single Component Core + Multicomponent Shell

In this design category, the sole purpose is to reinforce the shell component integrity by addition of complementary polymers. The added polymer could contribute to reinforcement either by having physical interactions with the shell polymer,^[^ ^106^ ^]^ or simply on account of its own resistant nature.^[^ ^35^ ^]^ For instance, electrospraying was used to encapsulate *Lactobacillus acidophilus* (*L. acidophilus*) in alginate, and it was subsequently coated with a composite of egg albumen (EA) and stearic acid (SA) via fluidized bed coating.^[^ ^107^ ^]^ Increasing in relative proportion of SA led to enhanced encapsulation efficiency, as well as significant improvement in viability of encapsulated cells exposed to the moist‐heat treatment (at 70 °C and 100% relative humidity for 30 min). This study highlighted the importance of physical interactions between multiple shell components (ionic bonds between SA and EA) in providing a better protection for the encapsulated probiotic.

**Figure 5:**
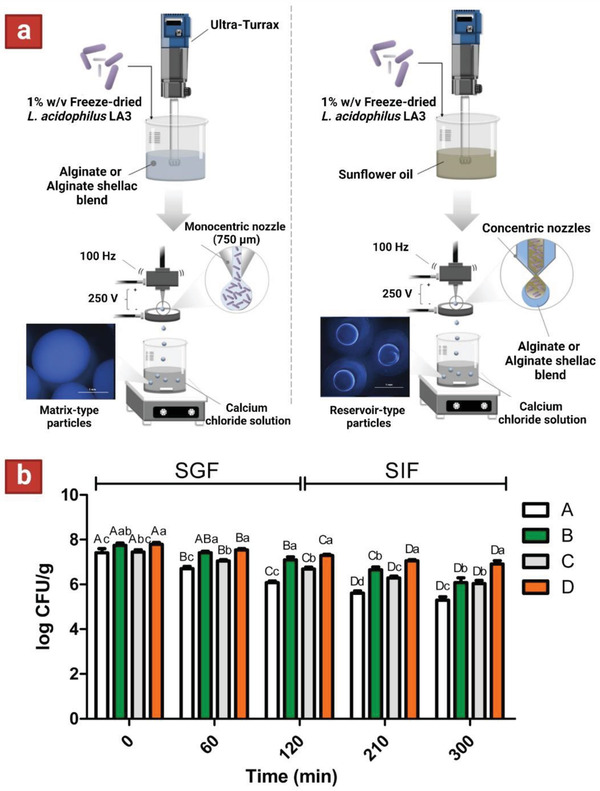
a) Schematic showing the corresponding technology and composition used for fabrication of single and core–shell microparticles, respectively. Scale bars on the microscopy images are 1 mm. b) Survival of encapsulated *Lactobacillus acidophilus* LA3 in simulated gastrointestinal fluids (120 min in simulated gastric fluid—SGF; 180 min in simulated intestinal fluid—SIF). Formulation A contains alginate and formulation B contains alginate–shellac. Formulation C is composed of alginate as the wall material and sunflower oil loaded with probiotics as the core, and formulation D is made of alginate–shellac as the wall material and sunflower oil loaded with probiotics as the core. Reproduced with permission.^[^ ^108^ ^]^ Copyright 2018, Elsevier.

Another approach is the application of resistant polymers in conjugation with the primary shell polymer. To this end, a co‐extrusion technique was used to encapsulate *L. acidophilus* using sunflower oil as the core and a blend of alginate–shellac as the shell material (**Figure** **5**a).^[^ ^108^ ^]^ After 60 days of storage at room temperature, the dried core–shell microparticles containing shellac showed higher cell viability when compared to ones without shellac. Also, after incubation in simulated gastrointestinal fluids (SGF — pH 1.8; SIF — pH 6.5), the microparticles containing shellac only showed slight reduction in cell viability, while formulation without shellac exhibited higher viability reduction (Figure 5b). Altogether, addition of shellac caused reduced porosity of the shell, as well as contributing acid‐resistant properties which led to improvements in cell viability both during storage and gastrointestinal conditions.

Overall, the depth of studies in the literature on “Single component core + multicomponent shell*”* structures is still limited, and more work needs to be done to further assess their potential in protecting the probiotics. However, the few existing studies proved the immense potential of this method to endow enhanced protection of the probiotics by simply using a composite approach for the shell material.

### 3.2 Three‐Layer Microparticles

Three‐layer microparticles can encompass several different compositions depending on their fabrication process. They can be made using either a water‐in‐oil‐in‐water (W_1_/O/W_2_) emulsification technique or via layer‐by‐layer self‐assembly. The W_1_/O/W_2_ contains an aqueous core (with/without a polymer) surrounded by a middle layer of oil, which is covered with an aqueous polymeric outer shell. The layer‐by‐layer, on the other hand, consists of an anionic/cationic polymeric core covered with an oppositely charged polymeric middle layer that is subsequently coated with a polymeric outer shell possessing an opposing charge in correspondence to the middle layer.

Generally, in W_1_/O/W_2_ microparticles the aqueous polymeric outer shell is not cross‐linked and as a result, these particles do not have long stability during storage and in gastrointestinal tracts.^[^ ^52^, ^58^, ^109^, ^110^, ^111^, ^112^ ^]^ Ionic cross‐linking was proposed to tackle this issue, in which the polymeric outer shell (W_2_) has been mixed with an oppositely charged polymer.^[^ ^51^, ^55^ ^]^

**Figure 6:**
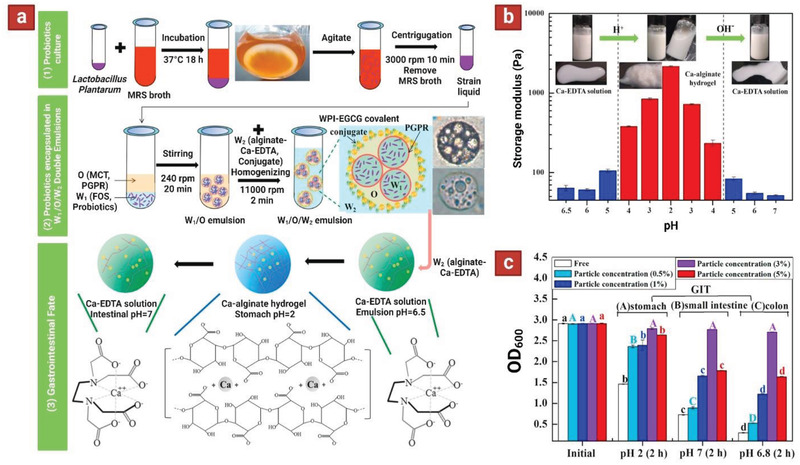
a) The process flowchart for encapsulation and colon‐targeted release of *Lactobacillus Plantarum* in W_1_/O/W_2_ double emulsions based on alginate–Ca–EDTA system. b) Effect of pH on the visual observations and storage modulus (*G*′) of W_1_/O/W_2_ double emulsions based on alginate–Ca–EDTA system. c) OD600 values of *Lactobacillus Plantarum* strain liquid encapsulated by W_1_/O/W_2_ double emulsions with different WPI‐EGCG covalent conjugate particle concentration (0.5, 1, 3, 5% w/v) during each step of the GIT digestion. Reproduced with permission.^[^ ^57^ ^]^ Copyright 2021, Elsevier.

In a notable study using a W_1_/O/W_2_ technique, three‐layered microparticles were fabricated with the following composition: *L. Plantarum* and fructooligosaccharides in the core; medium chain triglycerides oil and polyglycerol polyricinoleate emulsifier (PGPR) in the middle layer; and alginate–Ca–EDTA mixed with whey protein isolate‐epigallocatechin‐3‐gallate nanoparticles (WPI‐EGCG) in the outer shell layer (**Figure** **6**a).^[^ ^57^ ^]^ The middle oil phase (O) of double emulsion was used to resist bile salts and digestive enzymes in the small intestine. WPI‐EGCG nanoparticles were added as an amphiphilic additive with positive charge to facilitate ionic cross‐linking of alginate (W_2_). Additionally, Ca‐EDTA was incorporated to endow pH‐responsiveness to the alginate outer shell (W_2_). Under the acidic pH of the stomach, the ionic bonding between Ca^2+^ and alginate is encouraged; while under the neutral pH of the intestine, a strong chelation between EDTA and Ca^2+^ leads to disintegration of the cross‐linked alginate network (Figure 6b). The results showed that increasing PGPR concentration from 0.5% to 5% w/v decreased the droplet sizes nearly 43%, from 39 to 22 µm. On the other hand, with an increase in the inner oil fraction from 0.2 to 0.8 (W_1_/O), the droplet size increased almost 2.5‐fold, from 8 to 21 µm. Moreover, increasing WPI‐EGCG concentration from 1% to 5% w/v reduced the particle diameter almost 60%, from 48 to 19 µm, and all the formulations kept physically steady after 7 days of storage in room temperature. Remarkably, the probiotic viability in the W_1_/O/W_2_ double emulsion prepared with the optimal parameters (PGPR content of 3%, oil fraction of 0.6, and 3% WPI‐EGCG concentration) experienced no loss after full simulated GIT digestion (SGF‐pH 2, SIF‐pH 7, and simulated colonic fluid‐pH 6.8) (Figure 6c). Overall, this three‐layer formulation with pH‐sensitive alginate–Ca–EDTA and middle oil phase provided a colon‐targeted release carrier for the *L. Plantarum* with effective protection effect.

Layer‐by‐layer self‐assembly is a useful modality in the fabrication of three‐layer microparticles with sequentially opposingly charged polymers in each layer.^[^ ^64^, ^78^, ^79^, ^113^ ^]^ Alternatively, two‐layer microparticles fabricated through co‐extrusion could be further dip‐coated with a polymeric outer shell layer with opposite charge.^[^ ^40^, ^62^ ^]^ Regardless, the choice of biopolymer for the outer shell layer could be either a negatively charged polymer or a positively charged one. Accordingly, under acidic pH of the stomach, positively charged polymers are protonated while negatively charged polymers are deprotonated. This excess charge in the outer shell layer will contribute to stronger ionic interactions with the opposing polyelectrolyte in the middle layer, hence endowing acid resistant properties to the particles.

The encapsulation of probiotic *L. acidophilus* through layer‐by‐layer self‐assembly of polyelectrolytes chitosan (CHI) and carboxymethyl cellulose (CMC) was practiced to make three‐layer CHI–CMC–CHI microparticles.^[^ ^113^ ^]^ For uncoated cells, the number of viable cells dropped almost 27% after sequential freezing and freeze‐drying. On the other hand, the encapsulated cells only showed 8.4% viability losses under similar circumstances. Upon incubation in simulated gastrointestinal fluid, (SGF‐pH 2, followed by SIF‐pH 8) the number of viable cells declined almost 61% for uncoated formulations, while coated cells (CHI–CMC–CHI) only showed 12% reduction in viability.

**Figure 7:**
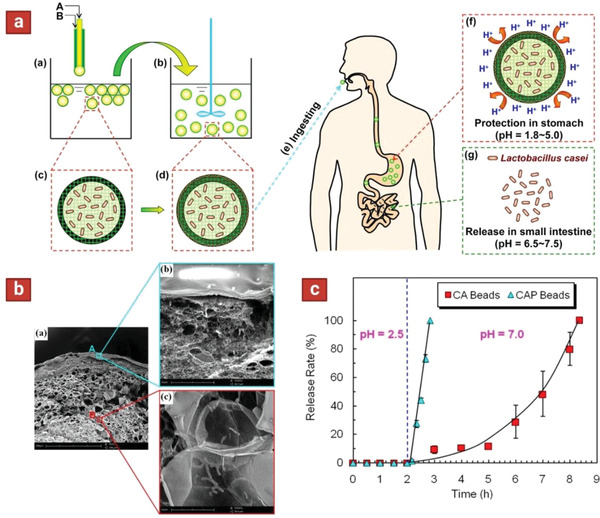
a) Schematic showing the fabrication of three‐layer microparticles and their pH‐responsive attributes for targeted release of cells in the intestine. First core–shell particles are fabricated using a co‐extrusion technique, and subsequently dip‐coated with protamine. A” is Na–alginate solution containing *Lactobacillus casei*, and “B” is pure Na–alginate solution. b) Cross section of the three‐layer microparticles using scanning electron microscopy (SEM). c) Release rate of *Lactobacillus* from two‐layer (CA) and three‐layer (CAP) microparticles after exposure to gastric acid (pH 2.5) and intestinal fluid (pH 7.0). Reproduced with permission.^[^ ^40^ ^]^ Copyright 2014, American Chemical Society.

Similarly, using co‐extrusion another report encapsulated *L. casei* in two‐layer microparticles (containing alginate in both core and the shell; CA) and subsequently immersed in positively charged protamine solution to yield alginate–alginate–protamine (CAP) three‐layer microparticles (**Figure** **7**a,b).^[^ ^40^ ^]^ It was hypothesized that in the acidic pH of the stomach, the diffusion channels in the second layer are delineated by choking the calcium–alginate networks with protonated protamine molecules; as a result, the gastric juice hardly diffuses across the CAP composite shell. To test this theory, Vitamin B_12_ (VB_12_) was incorporated in the formulations as a model solute and its diffusional permeability across the microparticles was tested in vitro in simulated gastric acid (pH 2.5). The diffusional permeability coefficient (*P* value) of VB_12_ across CAP shells was significantly lower than that of CA shells. The release rate of *L. casei* from CA and CAP microparticles was measured upon immersing in pH 2.5 gastric acid and then pH 7.0 intestinal fluid. In pH 2.5, no cells were released from both formulations. Yet, in pH 7.0 SIF, it only took about 50 min for the CAP particles to release all the encapsulated cells, as opposed to 380 min for the CA formulation (Figure 7c). These results demonstrate that the CAP microparticles exhibited much more efficient intestinal‐targeted delivery attributes than the CA formulation.

Overall, in three‐layer microparticles, a judicious choice of layering materials can endow properties such as pH‐responsiveness to the microparticles, which can be beneficial for their targeted delivery to intestine/colon (**Table** **2**). However, care must be taken during fabrication of these microparticles as multistep fabrication methods may affect leakage of some portion of encapsulated probiotics out of the microparticles, and hence causing a lower cell encapsulation yield in the formulations. In the case of W_1_/O/W_2_ microcapsules, the cell leakage can be controlled by adjusting the concentration of the emulsifier, the oil phase, and the W_2_ phase. In the same manner, for layer‐by‐layer self‐assembled microparticles, the concentration of each layer can be tuned to maintain the high encapsulation yield. Furthermore, combination of co‐extrusion with dip coating is another strategy for reducing the processing steps, hence enhancing the cell encapsulation yield. In conclusion, three‐layer microparticles can address some of the short‐comings of two‐layer formulations, particularly acid resistance, by simply adding an extra protection barrier. The smaller pores associated with these microparticles exclude larger enzyme molecules like pepsin and pancreatin, preventing proteolysis of the cells, while allowing smaller nutrient molecules to pass through. Perhaps in vivo testing of these formulations will further endorse their application in probiotic delivery.

**Table 2:** Summary of three‐layer microparticles for probiotic delivery

Fabrication technique	First layer component/s	Second layer component/s	Third layercomponent/s	Probiotic strain	Results	Ref.
W_1_/O/W_2_	MRS broth	Medium chain triglycerides oil + polyglycerol polyricinoleate	Poloxamer 407	*Lactobacillus reuteri*	Encapsulated formulation enhanced the viability of probiotic during cold storage, as compared to control. Upon simulated gastrointestinal conditions the viability decreased with a higher rate for control compared to encapsulated samples.	^[^ ^54^ ^]^
W_1_/O/W_2_	Ca cross‐linked alginate	Soybean oil + polyglycerol polyricinoleate	Bacterial cellulose	*Lactobacillus acidophilus*	High survival rate (84%) of encapsulated cells after exposure to simulated gastrointestinal conditions, compared to the free cells (undetectable level). Cells released from particles showed three times higher colon‐adhesion efficiency than that of free cells (Ex vivo everted gut sac model).	^[^ ^111^ ^]^
W_1_/O/W_2_	Fructooligosaccharides	Medium chain triglycerides oil + polyglycerol polyricinoleate	Ca‐EDTA cross‐linked alginate + whey protein isolate‐epigallocatechin‐3‐gallate	*Lactobacillus plantarum*	The probiotic viability in the W_1_/O/W_2_ double emulsion prepared with the optimal parameters experienced no loss after full simulated gastrointestinal digestion.	^[^ ^57^ ^]^
W_1_/O/W_2_	MRS broth	Corn oil + polyglycerol polyricinoleate	Gelatine + gum arabic	*Lactobacillus plantarum*	After exposure to simulated gastrointestinal conditions, viability of the encapsulated cells was 80.4% whereas it was only 25.0% for the free cells at 37 °C.	^[^ ^55^ ^]^
Layer‐by‐layer	Chitosan	Carboxymethyl cellulose	Chitosan	*Lactobacillus acidophilus*	Lower loss in cell viability for coated cells (8%) when compared to free cells (27%), exposed to sequential freezing and freeze‐drying. Significantly less reduction in cell viability (12%) after exposure to gastrointestinal conditions, compared to that of free cells (61%).	^[^ ^113^ ^]^
Extrusion + Layer‐by‐layer	Xanthan	Chitosan	Xanthan	*Lactobacillus acidophilus*	Higher cell viability after gastrointestinal conditions when compared to xanthan‐chitosan particles.	^[^ ^64^ ^]^
Extrusion + Layer‐by‐layer	Ca cross‐linked alginate	Chitosan	Methacrylic acid‐Methyl methacrylate Copolymer (1:2)	*Lactobacillus acidophilus* *or* *Lactobacillus plantarum*	Improved cell viability after exposure to both gastrointestinal conditions, and incorporation into yogurt.	^[^ ^79^ ^]^
Co‐extrusion + Dip coating	Alginate + fish oil	Ca cross‐linked alginate + pectin	Soy protein isolate	*lactobacillus plantarum*	Oil‐containing microparticles significantly improved the encapsulation efficiency of probiotics and resulted in highest viability of probiotics when exposed to simulated gastrointestinal conditions (92%).	^[^ ^62^ ^]^
Co‐extrusion + Dip coating	Alginate	Ca‐cross‐linked alginate	Protamine	*Lactobacillus casei*	The diffusional permeability coefficient (*P* value) was significantly lower for protamine‐coated particles compared to two‐layer particles. Protamine‐coated particles showed responsive release of cells after exposure to intestinal pH.	^[^ ^40^ ^]^

### 3.3 Four‐Layer Microparticles

To date, the pinnacle of complexity for multilayer microparticles is the four‐layer structure that provides the most protection for the encapsulated probiotics (**Table** **3**). Similar to the previous class, the four‐layer microparticles are fabricated either by using layer‐by‐layer self‐assembly,^[^ ^80^, ^114^, ^115^, ^116^ ^]^ or by combination of a microencapsulation technique with this modality.^[^ ^75^, ^117^ ^]^ *Lactobacillus pentosus* (LP) was encapsulated in a four‐layer microparticle via layer‐by‐layer approach using positively charged chitosan (CS) and negatively charged sodium phytate (SP).^[^ ^114^ ^]^ The influence of simulated gastrointestinal conditions on the viability of plain and encapsulated probiotics in two‐layer (CS/ SP)1‐LP and four‐layer (CS/SP)2‐LP structures was tested. After incubation in SGF (pH 1.5), the viability of cells in four‐layer (CS/SP)2‐LP formulations were almost fourfold and twofold higher than that in plain‐LP and two‐layer (CS/SP)1‐LP formulations, respectively. Similarly, after incubation in bile salts (pH 6.8), the number of viable bacteria in four‐layer (CS/SP)2‐LP was almost threefold and twofold higher compared to that in single‐layer plain‐LP, or two‐layer (CS/ SP)1‐LP formulations, respectively. Also, 30 days storage of formulations at 4 °C caused a 40% reduction in cell viability for plain‐LP, yet this value only declined 12% for four‐layer (CS/SP)2‐LP formulations. These results underline that the process of layer‐by‐layer encapsulation effectively improves the survival rate during the storage and through gastrointestinal tract. The viability reduction of probiotics encapsulated in four‐layer structures under both circumstances was attributed to low thickness of the coating layers being around a few hundred nanometers.

**Table 3:** Summary of four‐layer microparticles for probiotic delivery

Fabrication technique	First/Second/Third/Fourth layer component/s	Probiotic strain	Comments	Ref.
Layer‐by‐layer	Chitosan/ Sodium phytate/Chitosan/ Sodium phytate	*Lactobacillus pentosus*	Significantly higher cell viability after gastrointestinal conditions compared to two‐layer particles. Improved cell viability after 30 days of storage at 4 °C, compared to free cells.	^[^ ^114^ ^]^
Layer‐by‐layer	Chitosan/ Dextran sulfate/Chitosan/ Dextran sulfate	*Saccharomyces boulardii*	2 Logs and 3 Logs higher cell viability for encapsulated cells (compared to free cells) after freeze‐drying and exposure to gastrointestinal conditions, respectively.	^[^ ^80^ ^]^
Layer‐by‐layer	Chitosan/Block‐copolymer of poly(acrylic acid) and pluronic/Chitosan/Block‐copolymer of poly(acrylic acid) and pluronic	*Lactobacillus delbrueckii* subsp.* bulgaricus*	Four‐layer particles had the largest lag times but the highest growth rate. Lowest reduction in viability after gastrointestinal conditions.	^[^ ^115^ ^]^
Layer‐by‐layer	Chitosan/Alginate/Chitosan/Alginate	*Bacillus coagulans*	Showed major survival advantages against both acid and bile insults as compared to their plain, and single‐bilayer‐coated counterparts. Enhanced mucoadhesion provided by four‐layers coating led to growth advantages during the first 6 h (Ex vivo porcine small intestines and Intestine‐mimicking tissues from humans). Four‐layer coating exhibited significant survival advantages as compared to plain‐BC in the colon tissues (in vivo‐mice).	^[^ ^116^ ^]^
Emulsion + Layer‐by‐layer	Ca‐cross‐linked alginate/Chitosan/Alginate/Chitosan	*Lactobacillus rhamnosus* GG	In a custom‐made human gastrointestinal simulator system, the four‐layer particles boosted the metabolic function of cells in the small intestine, ileum, and colon by a factor of (2−3) × 10^5^ times, compared to free cells.	^[^ ^117^ ^]^
Extrusion + Layer‐by‐layer	Ca‐cross‐linked alginate/Protamine/Alginate/Protamine	*Escherichia coli*	Responsive release of cells to intestinal pH. Colon adhesion strength is enhanced by adding layer numbers (Ex vivo‐rat colon model). Highest survival advantages in the colon belonged to four‐layer microparticles (In vivo‐mice).	^[^ ^118^ ^]^

**Figure 8:**
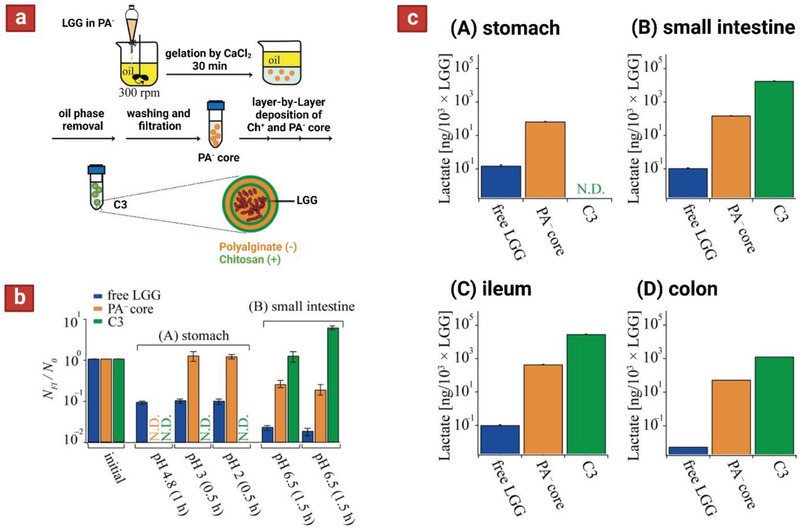
a) Schematic showing microencapsulation of *L. rhamnosus* Strain GG (LGG). Initially the cells were encapsulated in single alginate particles (using emulsification) and subsequently subjected to alternating coatings layers of chitosan and alginate via layer by layer deposition. b) Viability of cells released in the human gastrointestinal simulator. c) LGG lactate production after passage through each phase: stomach for 2 h (A), small intestine for 3 h (B), ileum for 3 h (C), and colon for 18 h (D). Reproduced with permission.^[^ ^117^ ^]^ Copyright 2018, American Chemical Society.

To address this issue, *L. rhamnosus* GG (LGG) was first encapsulated in alginate microparticles using emulsification. Subsequently, these particles were subjected to layer‐by‐layer deposition of chitosan–alginate–chitosan to reach four layers of coating (**Figure** **8**a).^[^ ^117^ ^]^

Free LGG, single alginate particles, and four‐layer microparticles were tested in vitro in fluids mimicking upper gastrointestinal environments (SGF‐pH 2, and SIF‐pH 6.5), and the viability of cells was measured. The results indicated that cells encapsulated in four‐layer structures fully maintained their viability after incubation in both environments. Furthermore, a custom‐made human gastrointestinal simulator system was used to assess the performance of formulations upon sequential passage through stomach, small intestine, ileum, and colon. The four‐layer microparticles released no probiotics, even after 2 h dwell in the stomach phase, and only started the cell release after 1.5 h incubation in the intestinal phase. Notably, in the beginning of intestinal phase the number of living LGG was close to the initial level *N*/*N*_0_ ≈ 1, which significantly increased after incubation of the sample for another 1.5 h in the intestine phase (*N*/*N*_0_ ≈ 5) (Figure 8b). This was assumed to be a result of LGG proliferation in the microparticles and their slow release over time. Last, with the aim of assessing metabolic function of LGG delivered to the ileum and colon, the concentration of lactate produced by LGG was measured in the simulator in different phases including: A) stomach, B) small intestine, C) ileum, and D) colon. For LGG in four‐layer microparticles, no lactate production was detected under the stomach condition (no release in the stomach). On the other hand, these microparticles boosted the metabolic function of LGG in the small intestine, ileum, and colon by a factor of (2−3) × 10^5^ times, compared to free LGG (Figure 8c). These data demonstrate that four‐layer formulation of LGG boosts not only the viability but also the metabolic functionality of probiotics throughout oral uptake, passage through the gastrointestinal tract, and delivery to the ileum and colon.

Similarly, an enzyme‐sensitive microparticle was constructed for colon‐targeted delivery of *Escherichia coli* (*E. coli*) to the colon.^[^ ^118^ ^]^ First, alginate microparticles are fabricated using electrostatic droplet extrusion, and subsequently coated with alternating layers of protamine and alginate via the layer‐by‐layer self‐assembly. The multilayer microcapsule can protect the probiotics in the stomach and disintegrate layer by layer under the action of trypsin in the intestine. The four‐layer microparticles exhibited minimal release at the gastric pH value but a burst release after 1 h at the intestinal pH value. The authors also showed the highest potential to protect the cells against simulated gastrointestinal conditions and bile salts. Upon ex vivo testing (using fresh rat colon model), the adhesion strength of cells is improved with the increase of the coating layer number. Last, in vivo colonization in mice found that highest survival advantages in the colon belonged to four‐layer microparticles. Moreover, the blood biochemistry and histological analysis demonstrated the safety of the microcapsule formulation.

Overall, four‐layer microcapsules provided a unique platform for targeted delivery of probiotics to the colon. This is mainly attributed to superior acid resistance of these formulations, resulting from the larger extent of ionic bonds associated with higher number of oppositely charged coating layers. However, to take full advantage of this property the thickness of the coating layers must be optimal to avoid their quick degradation. At the same time, the thickness cannot exceed a certain value, as it could prolong the lag time and interfere with the probiotic growth kinetics. In conclusion, design of four‐layer structures must take multiple factors into consideration to achieve optimal protection of the probiotics.

## 4 Applications of Multilayer Microencapsulated Probiotics

The prevalence and incidence of functional gastrointestinal conditions have been increasing worldwide and characterized as motility disturbances, dysbiosis, immunomodulatory changes, mucosal disruptions, and altered central nervous system processing.^[^ ^119^, ^120^, ^121^ ^]^ These conditions have both economic and social impacts with implications on global healthcare costs and impaired quality of life. Inflammatory bowel disease (IBD) and metabolic syndrome have the potential for better management using probiotic formulations. Statistically, the prevalence of individuals impacted by dyspepsia and irritable bowel syndrome range from 5.3–20.4% and 1.1–29.2% worldwide depending on age with some countries such as the US having rates as high as 35% for persons over 65.^[^ ^120^ ^]^ These conditions and their associated risk factors will be reviewed in the following section along with in vivo models that have been used with multilayer probiotic formulations.

**Figure 9:**
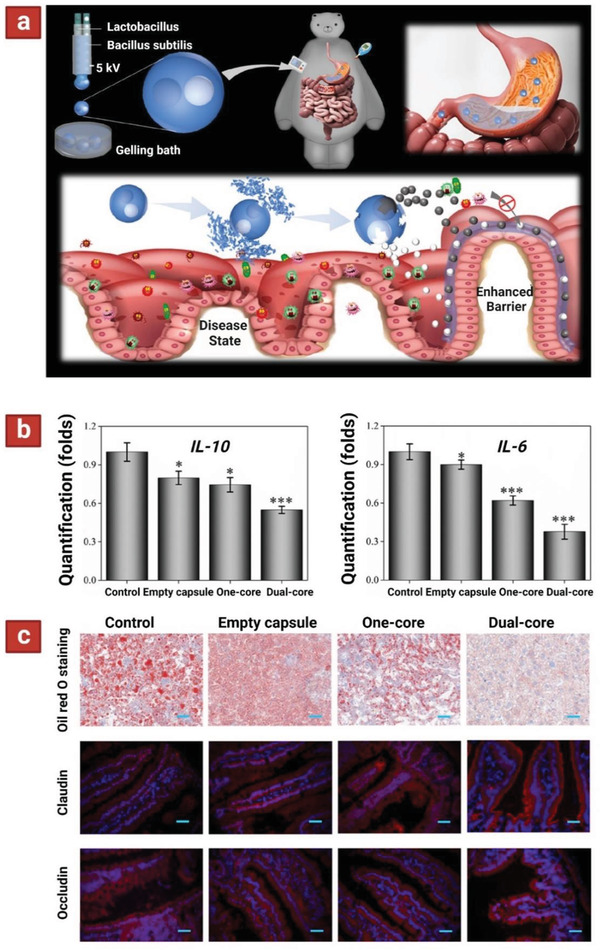
a) Schematic depicting the fabrication of probiotic‐encapsulated dual‐core microcapsules (top panel). These microparticles are fabricated using microfluidic chip containing two‐bore inner and outer capillaries. The goal is to simultaneously deliver two probiotics to the intestinal lumen (bottom panel). b) Quantification of IL‐10 and IL‐6 in intestine of mice treated with dual‐core microcapsule group, directly mixed probiotic microcapsule group (one‐core), empty capsule group, and a control group without any treatment. c) Therapeutic effect on preventing HFD‐induced MetS. Liver oil red O staining showing liver steatosis with different interventions. Intestinal immunofluorescence staining of gut barrier proteins, including claudin and occludin. Scale bars are 40 µm in oil red O staining and 50 µm in claudin and occludin stainings. Reproduced with permission.^[^ ^125^ ^]^ Copyright 2020, American Chemical Society.

Metabolic syndrome is characterized through a grouping of risk factors associated with the development of cardiovascular disease and type 2 diabetes.^[^ ^122^, ^123^ ^]^ Elevated serum lipid levels is a measurable factor that may lead to the development of cardiovascular disease, coronary heart disease, and atherosclerosis.^[^ ^123^ ^]^ First generation treatments for elevated serum lipid levels include drug therapy, dietary intervention, and exercise, however, it has been shown that probiotic organisms exert similar effects when administered to mice.^[^ ^124^ ^]^ A hamster model was setup using an induced metabolic syndrome hamster and feeding encapsulated *L. rhamnosus*, and *L. fermentum* produced via extrusion and embedded within an alginate core with a double layer of E‐polylysine and alginate over 6 weeks. Total serum cholesterol, LDL‐cholesterol and triglyceride levels were lower compared to high fat diet (HFD) controls by ≈32%, 41%, and 42%, respectively, which indicates the possibility to treat metabolic syndrome risk factors such as serum lipid levels.^[^ ^122^ ^]^ Similarly, using HFD hyperlipidemic mice to induce metabolic syndrome, this model was tested using a dual‐core multilayer probiotic formulation containing *Lactobacillus* and *B. subtilis* (**Figure** **9**a).^[^ ^125^ ^]^ First, probiotic bioactivity was investigated under digestive environment. Microspheres without the alginate shell and the dual‐core microcapsules were incubated in SGF. The microsphere group reacted quickly after encountering SGF and probiotic activity lost with time, particularly with a sharp decrease. However, in the dual‐core microcapsule group, the probiotics maintained 70% of their activity even after 60 min. These findings indicated that the alginate shell of the dual‐core microcapsules could effectively protect the activity of the probiotics. The slight drop of activity is attributed to the slow penetration of stomach acid in these microparticles.

Next, animal studies were carried out in mice with metabolic syndrome and results indicated that the dual‐core system for delivery of probiotics reduced the expression of two inflammatory cytokines in the intestine (IL‐6 and IL‐10; Figure 9b), and immunostaining of important intestinal barrier proteins (claudin‐1 and occludin) showed decreased intestinal hyperpermeability (Figure 9c). It is suggested that the interplay between the inflammatory cytokines and probiotics allows reduction in permeability of the intestinal wall and therefore reduced fat deposition within the liver and bloodstream. IL‐6 and IL‐10 are markers of both IBD and metabolic syndrome and can cause inflammation in the colon if continuously being produced. These results indicate the improvement of serum lipid levels and reduction of inflammatory markers through treatment with multilayer probiotic formulations and present data, suggesting a potential for improving human health.

**Figure 10:**
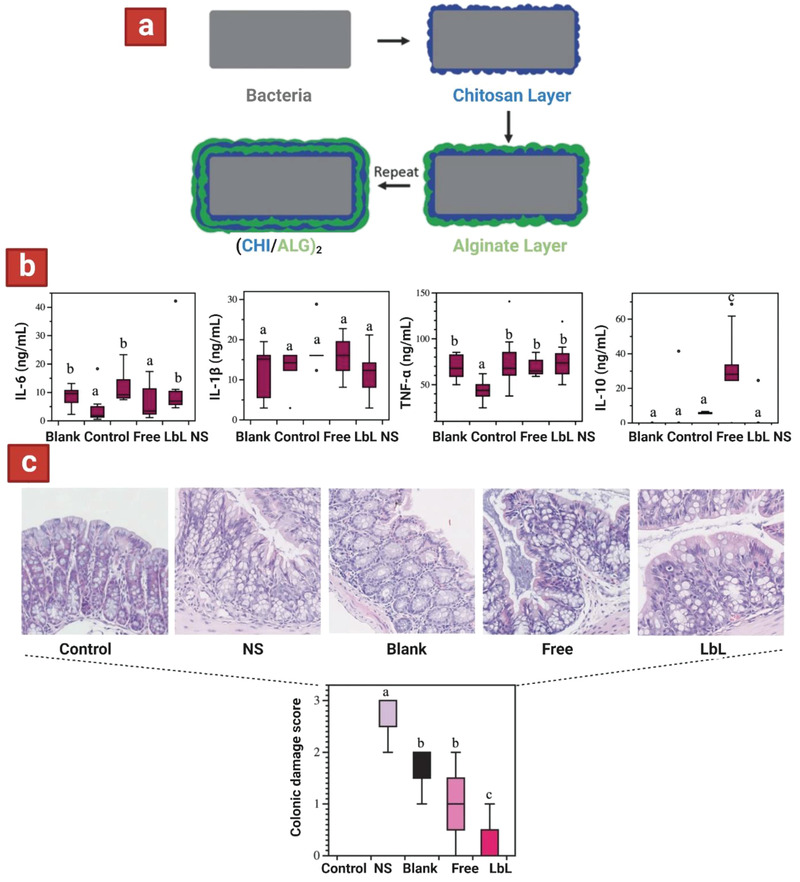
a) Schematic layer by layer templating of chitosan and alginate on probiotic. Reproduced with permission.^[^ ^116^ ^]^ Copyright 2016, Wiley‐VCH GmbH. b) Concentrations of inflammatory cytokines were measured in the plasma in DSS colitis‐induced mice. c) Histology images of colonic damage were taken, and colonic damage scores were measured in DSS colitis‐induced mice. Saline buffer (NS), chitosan and alginate mixture (Blank), nonencapsulated Li01 (Free), and LbL encapsulated Li01 (LbL). Reproduced with permission.^[^ ^121^ ^]^ Copyright 2021, Macmillan Publishers Ltd.

IBD is a disease with an increasing prevalence and incidence worldwide.^[^ ^121^, ^125^ ^]^ The conditions that are caused by IBD include ulcerative colitis and Crohn's disease which are often chronic with severe acute symptoms that can be instigated by genetic predispositions and environmental factors. To measure the impact of probiotics on IBD symptoms, the areas of focus are gut microbiota homeostasis, mucosal barrier integrity, immune response, defense against invading pathogens, and prevention of chronic inflammation. Dextran sulfate sodium (DSS)‐induced acute colitis mice were treated using *L. salivarius* (Li01) encapsulated using layer by layer (LbL) techniques of chitosan and alginate bilayers to form a multilayer coating for 14 days (**Figure** **10**a).^[^ ^121^ ^]^ Initially, the survivability of free Li01 and LbL Li01 to SGFs and SIFs was quantified. Enhanced resistance in SGF was observed in encapsulated Li01 (Figure 2a,c); after incubation for 20 min, viable free Li01 decreased from 10.2 log CFU/mL to 6.3 log CFU/mL compared to a decrease of 1.2 log CFU/mL among LbL Li01. In SIF, the number of viable, free Li01 cells was quickly reduced from 10.2 log CFU/mL to 4.0 log CFU/mL in 20 min. With confocal microscopy, most free Li01 cells were found to be dead in SGF and SIF whereas most of the LbL encapsulated cells appeared to be viable. After incubation for 2 h, no viable cells could be detected among the free Li01 either with viable count or microscopy. Yet, for LbL Li01, the total reduction was 3 log CFU/mL and the viable number of cells remained above 6 log CFU/mL. Subsequently, LbL Li01 were tested in DSS‐induced acute colitis mice and the inflammatory factors in the mice were measured in the plasma. Post‐treatment with encapsulated Li01 was associated with low levels of the pro‐inflammatory cytokines (including IL‐6, IL‐1*β*, and TNF‐*α*) and significant higher levels of anti‐inflammatory cytokine (IL‐10) compared to other groups, suggesting the superiority of encapsulated Li01 in facilitating colonic epithelial amelioration (Figure 10b). The histological samples also revealed significantly lower damage scores in mice treated with encapsulated Li01 compared to mice fed with other treatments, showing the potential for encapsulated Li01 to promote rapid recovery of the colonic epithelium (Figure 10c). Similarly, an alginate–chitosan LbL assembly encapsulated *E. coli* was used to treat 2,4,6‐trinitrobenzene sulfonic acid‐induced colitis model rats and inflammatory markers were examined.^[^ ^126^ ^]^ Treatment also reduced IL‐6, IL‐10, and TNF‐*α* compared to untreated models. Furthermore, histological analysis showed reduced tissue damage and inflammatory cell penetration compared to nontreated groups. These two models indicate the potential for multilayer probiotic formulations to reduce inflammatory markers associated with IBD through reduction of interleukins and TNF‐*α*. In another instance, chitosan‐coated alginate microcapsule loaded with in situ synthesized barium sulfate (CA/BaSO4 microcapsule) were used for oral *Bifidobacterium* delivery and real‐time X‐ray computed tomography imaging.^[^ ^127^ ^]^ Initially, the pH 2.5 simulated gastric acid was chosen as the model gastric acid to investigate the protection effect of CA/BaSO_4_ microcapsules on *Bifidobacterium*. Viability of free *Bifidobacterium* and *Bifidobacterium* encapsulated by CA/BaSO_4_ microcapsules were evaluated after being immersed in pH 2.5 gastric acid for 1 and 2 h. The survival of naked *Bifidobacterium*, and CA/BaSO_4_ microcapsule‐loaded *Bifidobacterium* were 36.1% and 55.7%, respectively, after 1 h of immersion. The same tendency appeared after 2 h of immersion, and the survivals rates were 19.8% and 44.3%, respectively. The results indicated that CA/BaSO_4_ microcapsules offered better protective effect. Next, to validate the advantage of CA/BaSO4 microcapsules in oral administration, DSS‐induced mice colitis model was chosen as the animal model. After 11 days of treatment, all DSS‐colitis mice showed a significant reduction in their colon length compared with the control groups. However, the colon length was gradually restored when treated with CA/BaSO_4_ microcapsules encapsulated with 2.4 × 109 CFU *Bifidobacterium*. Also, CA/BaSO4 microcapsules showed good visibility under X‐ray after they were fed to the mice, which is beneficial not only for observing the *Bifidobacterium* migration in the digestive tract but also for evaluating the engraftment of *Bifidobacterium* in the intestine.

Orally administered probiotics are generally known to be beneficial for alleviation of gastrointestinal conditions due to their ability to reestablish gut microbiota homeostasis, restore gut barrier integrity, modulate immune responses, protect against invading pathogens, and prevent chronic inflammation. However, therapeutic efficacy of probiotics over these conditions is highly reliant on their function in the intestine. This means they need to remain viable during storage and gastrointestinal transit. However, probiotics’ viability and activity are susceptible to environmental factors such as oxygen, gastric acid, and bile salts. Moreover, therapeutic efficacy may require promotion of colonization as the probiotics may also face colonization resistance from commensal bacteria. Herein, we have highlighted multilayer microencapsulated probiotics implemented in animal models with improved performance (compared to their pristine counterparts) for treating various gastrointestinal conditions. These multilayer systems contributed to amelioration of gastrointestinal conditions by significantly enhancing the survival of probiotics and endowing better mucoadhesive properties. Although the published data indicate the potential for multilayer systems in the clinical treatment of gastrointestinal conditions, further research is still required to better understand their impact.

## 5 Summary and Future Directions

The past decade has witnessed widespread advances in development of microencapsulated probiotics for delivery to the colon. Emergence of new fabrication technologies coupled with discovery of new materials has led to the development of new multilayer microparticles that offers extra protection to probiotics during manufacturing, storage, and gastrointestinal transition. Specifically, co‐extrusion techniques (via vibrational technologies, or microfluidic chips) allowed fabrication of multilayer microparticles with precise control over the composition and dimension of each layer. On the other hand, emulsion techniques provided a simple and cost‐effective approach to fabricate multiplayer microparticles (via W_1_/O or W_1_/O/W_2_ phases), at the cost of losing dimensional control over each layer. Notably, co‐extrusion of emulsion phases through microfluidic chips with multiple entry channels has helped to rectify the dimensional control in these systems. Additionally, coating technologies (via dip coating or layer‐by‐layer deposition) have been employed in both singular and plural manner (coupled with pre‐microencapsulated particles) to add extra protective layers on top of probiotic formulations. Despite the simplicity of coating technologies methods, they are only capable of yielding thin coating layers (10–100 nm), which could be detrimental to the probiotic protection being insufficient or inferior to other processes. Regardless, there is growing evidence for two‐, three‐, and four‐layer microparticles as promising platforms for delivery of probiotics to the colon.

The composition of layers in the corresponding multilayer microparticles plays a distinct role in protection of the probiotics. Accordingly, each layer contains a primary polymeric or oily phase, to which additional materials can be added to enhance the probiotic protection. In fact, both the intra‐ and inter‐layer interactions further contribute to probiotic protection by yielding a higher degree of cross‐linking in the polymeric networks. For instance, the addition of a complementary polymer that interacts with the primary polymer, through ionic and/or hydrogen bonds, is an approach to achieve this purpose. On the other hand, opposingly charged primary polymers in adjacent layers ionically interact with each other to enhance the layers integrity. Such interactions are further tuned by adjusting the number of charged functionalities within each polymeric backbone, via chemical modifications and/or copolymerization. The given strategies have been shown useful in protecting the probiotics against manufacturing and storage conditions; however, further consideration is required to bestow gastrointestinal resistance to the microparticles. To achieve this task, the outermost layer of the microparticles is instrumental, by incorporating materials that resist the acidic gastric fluid, as well as bile salts and enzymes in the intestinal fluid. To this end, polycationic or polyanionic polymers are used that undergo protonation or deprotonation (respectively) in acidic pH of the stomach, hence reinforcing ionic interactions amongst the layers in a pH‐sensitive manner. Moreover, addition of extra cross‐linking agents and insoluble compounds such as shellac show benefits in enhancing gastrointestinal resistance. Generally, multilayer microparticles have shown superior performance in protecting probiotics both in vitro and in vivo, when compared to the single layer microparticle counterparts. Particularly, multilayer microencapsulated probiotics have been successfully used in multiple animal models to alleviate conditions such as metabolic syndrome and IBDs. This evidence is testament to the immense potential of multilayer microparticles in enhancing therapeutic efficacy of the probiotics and any other viable therapeutics for colon targeted delivery for the treatment of a specific disease.

The ever‐evolving field of probiotics will benefit from identification of new biopolymers and technologies to improve the performance of multilayer formulations. Cross‐linked polymers, such as photo‐cross‐linkable hydrogels, can provide protection against digestive enzymes and gastric juice in the stomach.^[^ ^128^ ^]^ Gastric acid resistance can also be helped by incorporating additives such as antacid agents^[^ ^129^ ^]^ and zwitterionic surfactants (such as lecithin).^[^ ^10^ ^]^ Moreover, pH‐dependent and colonic microbiota‐triggered biopolymers remain as highly promising approaches for achieving intestinal regions via oral route. The combination of these strategies could potentially yield a system with superior storage viability and gastrointestinal protection, as well as targeted delivery to the colon. In addition, several reports highlighted the utilization of composite approach to fabricate multilayer structures that contained more resistant shell materials, such as silica^[^ ^130^ ^]^ and sporopollenin (extracted from natural pollen),^[^ ^131^, ^132^ ^]^ to create more effective multilayer particles for probiotic encapsulation.

New advance technologies for microencapsulation enable simultaneous fabrication of multilayer that minimize complexity and endow higher level of control over each layer's composition. For instance, a microfluidic chip with complex design allowed fabrication of three‐layer microparticles with designated composition in each layer to endow pH stimulus‐responsive release of a therapeutic in the colon.^[^ ^133^ ^]^ Indeed, advanced microfluidic systems can allow fabrication of microparticles with multiple core compartments, which has been shown useful in simultaneous encapsulation of multiple probiotic strains.^[^ ^125^ ^]^ Features like this are beneficial when considering that most commercial probiotic products contain multiple bacterial strains, which could have competing effect amongst each other. There is untapped potential for encapsulated microparticles to codeliver probiotics and other therapeutics. Such a combinatorial approach could yield synergistic benefits.^[^ ^134^ ^]^

To conclude, multilayer microparticles could be an effective strategy for providing a higher level of protection for the encapsulated probiotics, and to increase the effectiveness of these therapeutics in biomedicine. However, care must be taken as increasing design complexity will inevitably escalate manufacturing costs and regulatory procedures to demonstrate safety and efficacy of the formulation. Hurdles for product development include premature release/degradation, nonspecific absorption, microorganism viability, and clearance time. Strong mucoadhesiveness provided by polymeric particulate systems may also lead to nonspecific adhesion in GIT. While engineering more complex solutions may overcome these hurdles, this complexity may impact on the feasibility of clinical trials that are necessary to validate the health impacts of such products. Thus, the final multilayer biopolymeric microparticles may represent a compromise between biological efficacy, engineering, and medical practicality. Ultimately, Given the rise of nutraceutical products in the market, further research is still needed to ensure that these new formulations are effective in protecting the probiotics during actual food production, packaging, storage, and transport. Furthermore, to expand the biomedical application of these products researchers need to better characterize the physiological or pathological pathways that can be modulated by probiotics. Ultimately, the probiotic is aimed to be consumed in the human body, and consequently high throughput testing methods that closely mimic the corresponding gastrointestinal conditions are useful in determining the optimal formulation of microparticles. All in all, there is still room for improvements in both design and testing of multilayer formulations, and future research on this topic will be crucial to make these platforms a game changer in the field of probiotic delivery.

## Conflict of Interest

The authors declare no conflict of interest.
